# Variations in the branching pattern of the internal iliac artery and its implications in trauma and surgery – a South Indian cadaveric study

**DOI:** 10.1590/1677-5449.202400752

**Published:** 2025-03-31

**Authors:** Satheesha Nayak Badagabettu, Ashwini Aithal Padur, Surekha Devadasa Shetty

**Affiliations:** 1 Manipal Academy of Higher Education, Manipal, Karnataka, India.

**Keywords:** internal iliac artery, iliolumbar artery, obturator artery, pelvis, vascular variation, artéria ilíaca interna, artéria ileolombar, artéria obturatória, pelve, variação vascular

## Abstract

**Background:**

The internal iliac artery (IIA) frequently shows variations in its branching pattern. Knowledge of its variations is helpful during gynecological and orthopedic surgical procedures.

**Objectives:**

To observe the branching pattern of IIA in the human pelvises and discuss its clinical implications.

**Methods:**

The study was conducted on 80 male hemipelvises (40 left halves and 40 right halves). The pelvic halves were obtained by making mid-line saw cuts through formalin embalmed adult human cadavers aged approximately 50-80 years. The IIA were dissected and cleaned. Variations of the internal iliac artery and its branches were noted. Relevant photographs were taken. Results were expressed as percentages.

**Results:**

Variations in the branching pattern were observed in 49 (61%) hemipelvises (right: 21, left: 28). Variations were more common (48%) in the branching pattern of the anterior division of IIA than the posterior division (20%). Variations of the main trunk were observed in 29% of cases. In 3% of cases, the IIA did not divide into two divisions. Among the individual branches, the iliolumbar artery showed variations in 29% of cases and the obturator artery in 25%. A common trunk of the internal pudendal and middle rectal arteries was found in 24% of cases and variations of the inferior gluteal artery were seen in 18% of cases.

**Conclusions:**

The study showed a high rate of occurrence of variant IIA branching patterns. Understanding the anatomical variations of the IIA and its branches is essential to minimize intraoperative blood loss and other complications during pelvic surgeries.

## RESUMO

**Contexto:**

A artéria ilíaca interna (AII) frequentemente apresenta variações em seus padrões de ramificação. Conhecer suas variações é útil durante procedimentos cirúrgicos ginecológicos e ortopédicos.

**Objetivos:**

Observar o padrão de ramificação da IIA na pelve humana e discutir suas implicações clínicas.

**Métodos:**

O estudo foi realizado em 80 hemipelves masculinas (40 hemipelves esquerdas e 40 hemipelves direitas). As hemipelves foram obtidas através de cortes com serra na linha média em cadáveres humanos adultos de aproximadamente 50-80 anos de idade embalsamados em formaldeído. As AIIs foram dissecadas e limpas. Foram observadas as variações da AII e seus ramos. Obtiveram-se fotografias relevantes. Os resultados foram expressos em porcentagem.

**Resultados:**

Foram encontradas variações no padrão de ramificação em 49 (61%) das hemipelves (direita: 21, esquerda: 28). As variações foram mais comuns (48%) no padrão de ramificação da divisão anterior da AII do que na sua divisão posterior (20%). Variações no tronco principal foram observadas em 29% dos casos. Em 3% dos casos, a AII não estava dividida em duas divisões. Com relação aos ramos individuais, a artéria ileolombar apresentou variações em 29% dos casos, e a artéria obturatória em 25%. Foi encontrado um tronco comum entre as artérias pudenda interna e retal média em 24% dos casos, e variações na artéria glútea inferior foram observadas em 18% dos casos.

**Conclusões:**

O estudo demonstrou uma alta ocorrência de variações no padrão de ramificação da AII. Entender as variações anatômicas da AII e seus ramos é essencial para minimizar a perda sanguínea intraoperatória e outras complicações durante cirurgias pélvicas.

## INTRODUCTION

The internal iliac artery (IIA) begins as a terminal branch of the common iliac artery in front of the sacroiliac joint. After a short course, it divides into anterior and posterior divisions at the level of the superior border of the greater sciatic foramen. Branches of its anterior division provide territorial supply to the pelvic viscera, the perineum, the gluteal region, and the adductor region of the thigh. The lower posterior abdominal wall, posterior pelvic wall, and gluteal region are all supplied by branches from its posterior division.^1^ Because of its location and wide region of supply, the IIA is extremely important surgically. The medical literature commonly mentions variations in the IIA’s vascular pattern due to embryological alterations. Since most of the organs in the pelvic cavity are supplied by branches of the IIA, this artery is thought to be directly related to the occurrence, development, and management of pelvic illnesses.^2^ Knowledge of anatomical variations of the IIA and its branches is helpful during pelvic surgery for vascular surgeons.

One of the life-saving operations for intractable pelvic bleeding is internal iliac artery ligation, which is also used to treat severe obstetric and pelvic haemorrhage.^3^ Surgeons must investigate the IIA and its pattern of division and branching in the treatment of pelvic neoplasms to ensure the safety of any interventional procedure and prevent untargeted embolization.^4^ Radiologists must be familiar with IIA variants to interpret pelvic area angiograms correctly. It is crucial to comprehend the anatomical course of the IIA in patients with pelvic fractures. According to clinical assessments, the internal iliac artery, external iliac artery, obturator artery, and aberrant obturator artery are the most often injured arteries.^5^

The current study is focused on observing the branching pattern of IIA in the pelvis and comparing the findings with previous studies. We discuss the morphological significance of the IIA and the clinical importance of its branches in the fields of surgery and interventional radiology.

## METHODS

We conducted this study on 80 pelvic halves obtained from the Department of Anatomy. The pelvic halves were obtained by making mid-line saw cuts through formalin embalmed adult human male cadavers aged approximately 50-80 years. No medical histories were available on these cadavers. This study is in compliance with the Helsinki Declaration and with local ethical guidelines. The internal iliac arteries (IIA) were dissected and cleaned. Variations of the IIA and its branches were noted. Relevant photographs were taken. Results were expressed as percentages.

## RESULTS

**Figure 1:**
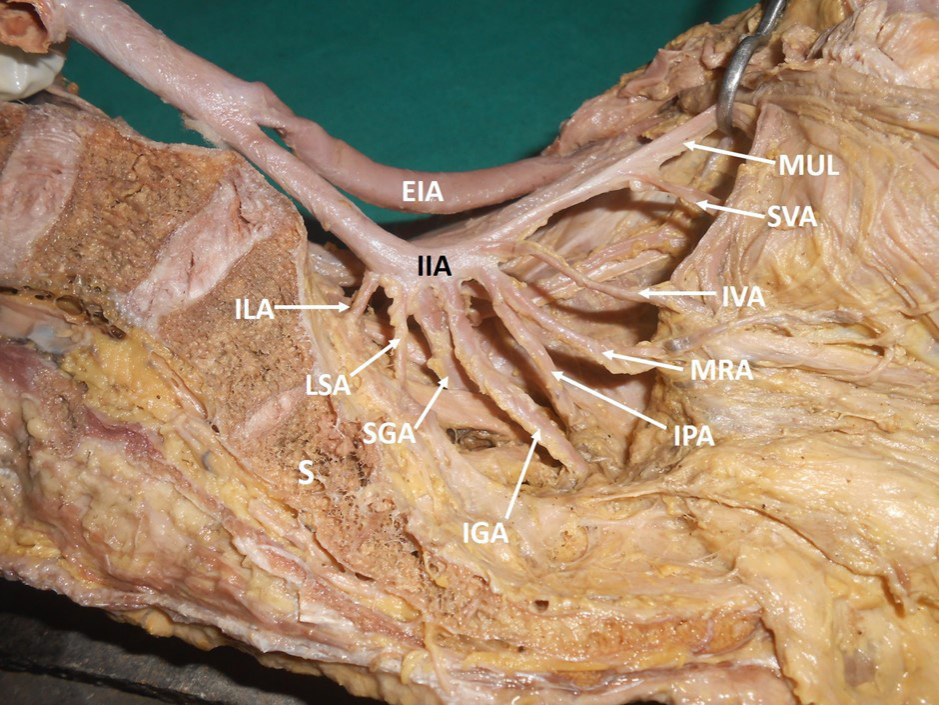
Dissected left hemipelvis showing the internal iliac artery giving off all its branches directly, without dividing into anterior and posterior trunks. IIA = internal iliac artery; EIA = external iliac artery; MUL = medial umbilical ligament; SVA = superior vesical artery; IVA = inferior vesical artery; MRA = middle rectal artery; IGA = inferior gluteal artery; IPA = internal pudendal artery; SGA = superior gluteal artery; LSA = lateral sacral artery; ILA = iliolumbar artery; S = sacrum.

Of the 80 hemipelvises studied, 40 belonged to right side and 40 to left side. 31 hemipelvises (right: 19, left: 12) did not show any variations of the IIA (39%), while variations in branching pattern were observed in 49 (61%) hemipelvis (right: 21, left: 28). Among the variations, 48% of variations were observed in the anterior division of the IIA (right: 22, left: 16), 20% variations were in the posterior division of the IIA (right: 6, left: 10), and 29% were in the main trunk of the IIA (right: 12, left: 11). In 3% (right: 1, left: 2), there was no division of the IIA. All of the branches arose from a single arterial trunk (Figure 1).

**Figure 2:**
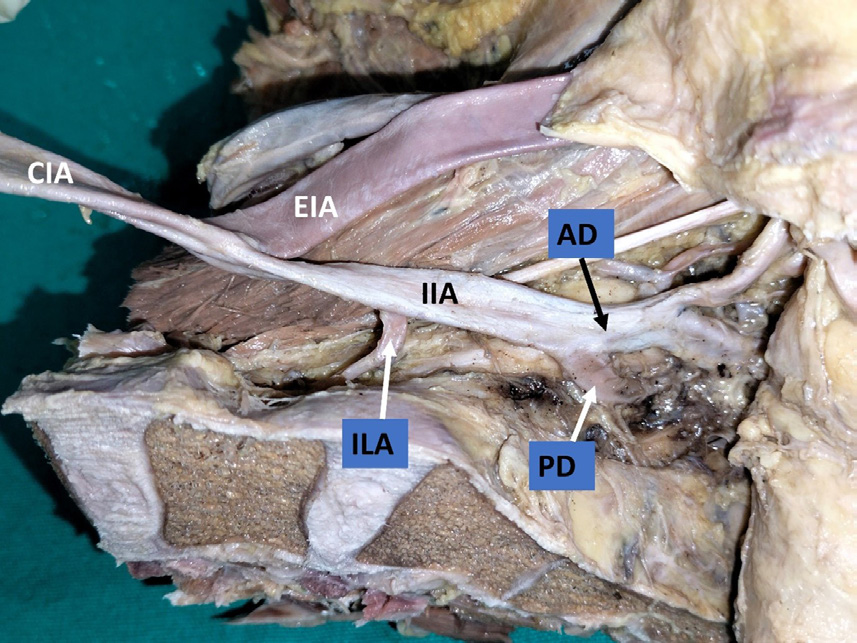
Dissected left hemipelvis showing origin of the iliolumbar artery from the main trunk of the internal iliac artery. CIA = common iliac artery; IIA = internal iliac artery; EIA = external iliac artery; AD = anterior division; PD = posterior division; ILA = iliolumbar artery.

**Figure 3:**
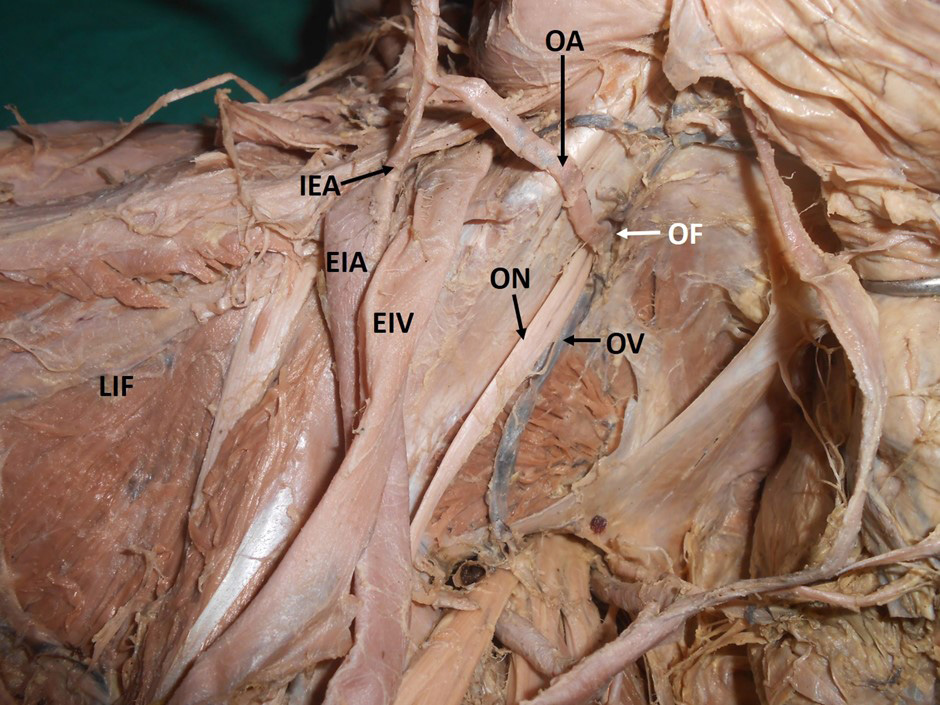
Dissected left hemipelvis, showing the abnormal obturator artery arising from the inferior epigastric artery. LIF = left iliac fossa; EIA = external iliac artery; EIV = external iliac vein; IEA = inferior epigastric artery; OA = obturator artery; ON = obturator nerve; OV = obturator vein; OF = obturator foramen.

**Figure 4:**
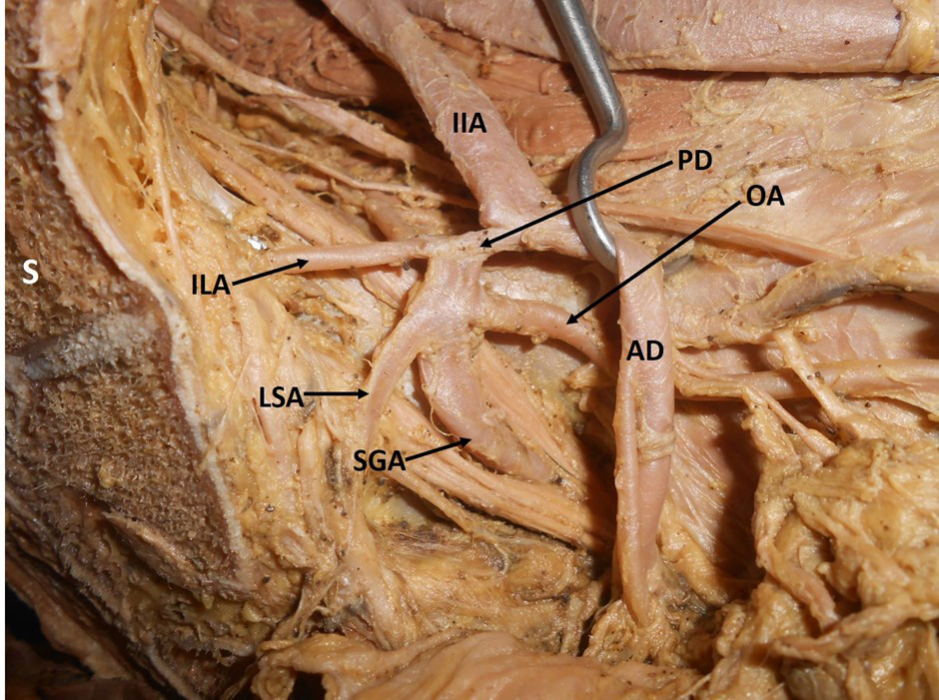
Dissected left hemipelvis showing origin of the obturator artery from the posterior division of the internal iliac artery. IIA = internal iliac artery; AD = anterior division; PD = posterior division; SGA = superior gluteal artery; LSA = lateral sacral artery; ILA = iliolumbar artery; OA = obturator artery; S = sacrum.

**Figure 5:**
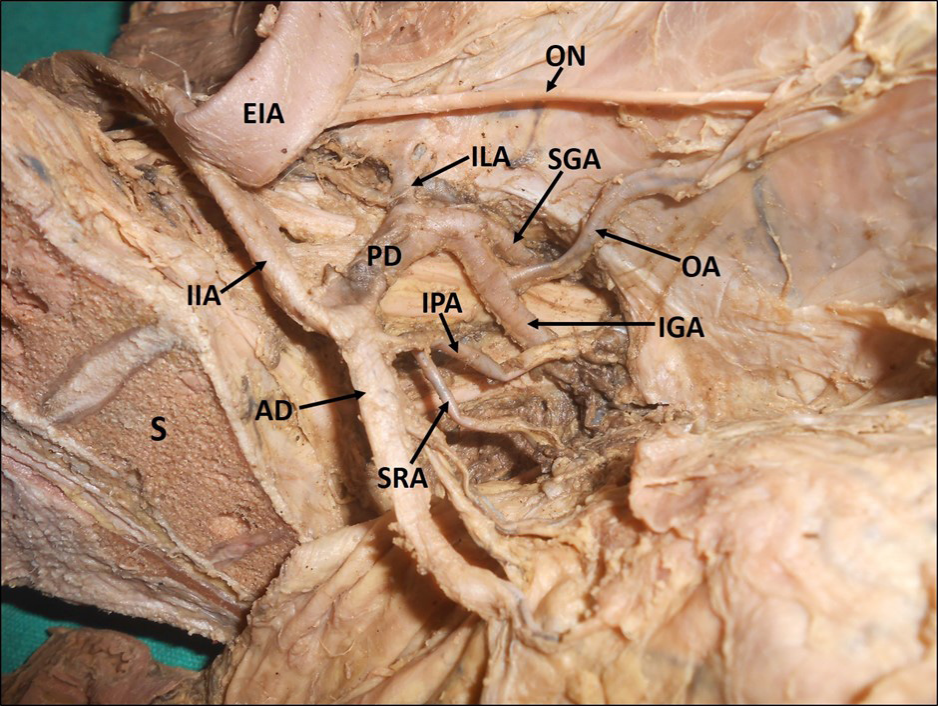
Dissected left hemipelvis showing origin of the obturator artery from the inferior gluteal artery (inferior gluteal artery is a branch of the posterior division of the internal iliac artery here) forming the corona mortis. IIA = internal iliac artery; EIA = external iliac artery; AD = anterior division; PD = posterior division; SRA = Sub-branch middle rectal artery; IGA = inferior gluteal artery; OA = obturator artery; ON = obturator nerve; IPA = internal pudendal artery; SGA = superior gluteal artery; ILA = iliolumbar artery; S = sacrum.

When variations related to the origin of the individual branches were observed, it was noted that the most frequent variation was the origin of the iliolumbar artery from the main trunk of the IIA (29%; right: 12, left: 11) (Figure 2) (Table 1). The next most frequent variation encountered was a variant origin of the obturator artery (25%; right: 8, left: 12). The obturator artery took its variant origin from other arteries like the external iliac artery, inferior epigastric artery (Figure 3), posterior division of the IIA (Figure 4), or the gluteal arteries (Figure 5), forming the corona mortis.

**Table 1:** Variations of the branches of the IIA.

Branches (% occurrence)	Right (n)	Left (n)
Iliolumbar artery (29%)	12	11
Obturator artery (25%)	8	12
Common trunk of internal pudendal artery and middle rectal artery (24%)	9	10
Inferior gluteal artery (18%)	6	8
Superior gluteal artery (4%)	2	1

**Figure 6:**
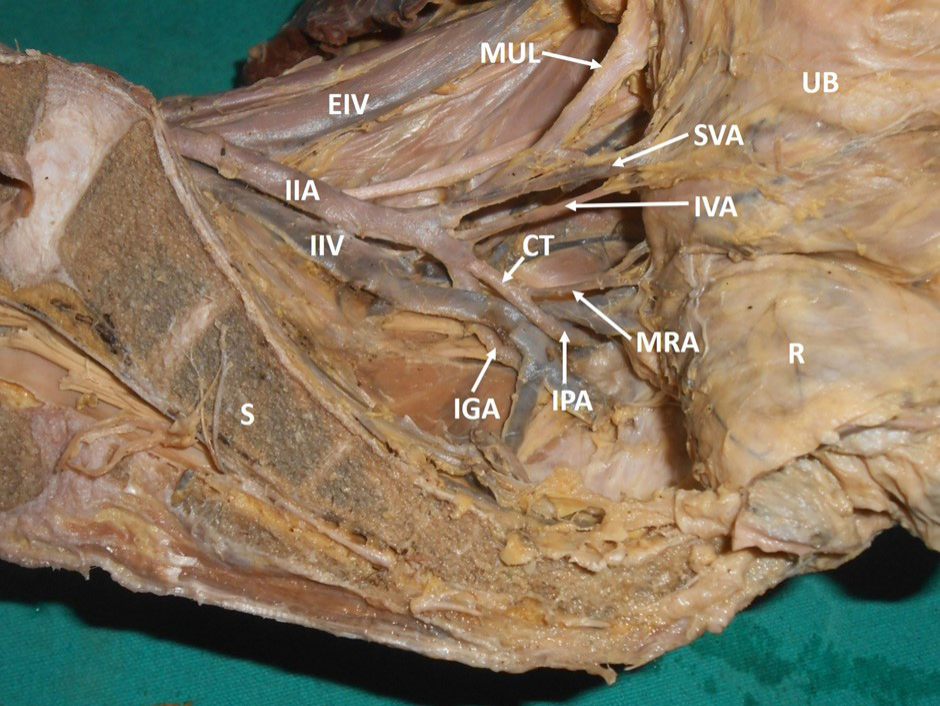
Dissected left hemipelvis showing a common trunk of the internal pudendal artery and middle rectal artery. IIA = anterior division of internal iliac artery; IIV = internal iliac vein; MUL = medial umbilical ligament; SVA = superior vesical artery; IVA = inferior vesical artery; MRA = middle rectal artery; IGA = inferior gluteal artery; IPA = internal pudendal artery; CT = common trunk of origin of internal pudendal and middle rectal arteries; EIV = external iliac vein; UB = urinary bladder; R = rectum; S = sacrum.

**Figure 7:**
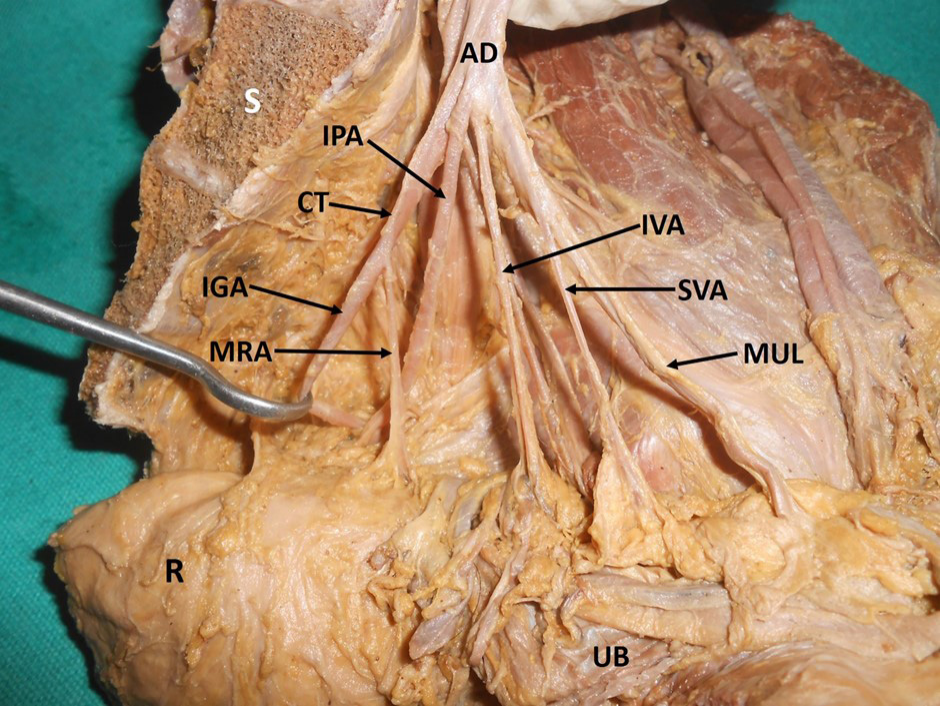
Dissected left hemipelvis showing a common trunk of the inferior gluteal artery and middle rectal artery. AD = anterior division of internal iliac artery; MUL = medial umbilical ligament; SVA = superior vesical artery; IVA = inferior vesical artery; IPA = internal pudendal artery; CT = common trunk of origin of the inferior gluteal and middle rectal arteries; IGA = inferior gluteal artery; MRA = middle rectal artery; UB = urinary bladder; R = rectum; S = sacrum.

**Figure 8:**
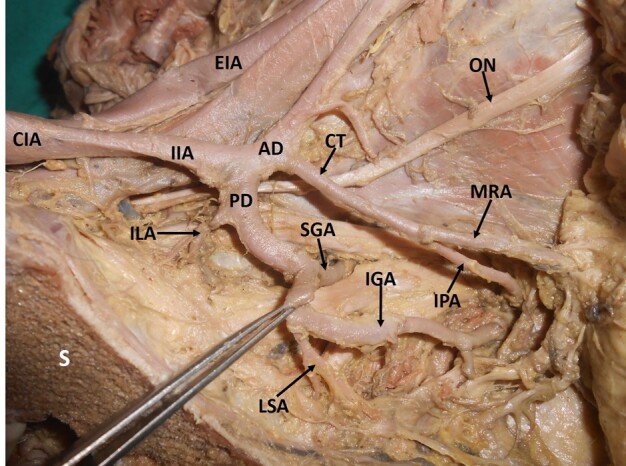
Dissected left hemipelvis showing the origin of inferior gluteal artery from the posterior division of the internal iliac artery. CIA = common iliac artery; IIA = internal iliac artery; EIA = external iliac artery; AD = anterior division; PD = posterior division; SGA = superior gluteal artery; IGA = inferior gluteal artery; CT = common trunk of origin of the middle rectal and internal pudendal arteries; MRA = middle rectal artery; IPA = internal pudendal artery; ON = obturator nerve; ILA = iliolumbar artery; LSA = lateral sacral artery; S = sacrum.

**Figure 9:**
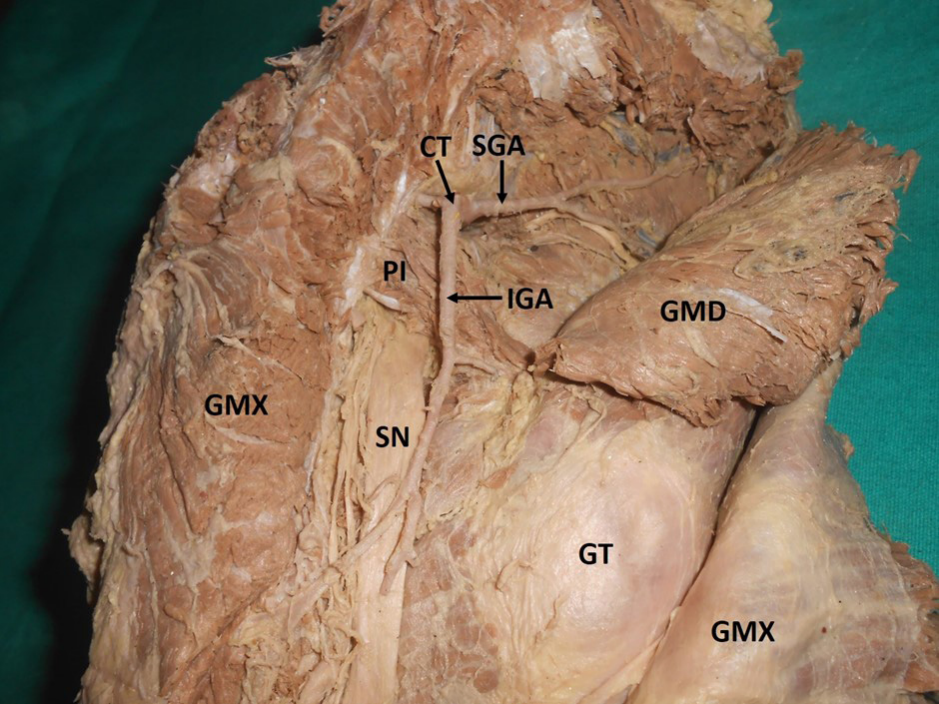
Dissected right gluteal region, showing a common trunk of the superior and inferior gluteal arteries. GMX = gluteus maximus; GMD = gluteus medius; GT = greater trochanter; SN = sciatic nerve; PI = piriformis; IGA = inferior gluteal artery; SGA = superior gluteal artery; CT = common trunk.

A common trunk of the internal pudendal artery and middle rectal artery was observed in 24% of specimens (right: 9, left: 10) (Figure 6). Absence or variant origin of the inferior gluteal artery from the posterior division of the IIA was observed in 18% (right: 6, left: 8) of the hemipelvises (Figure 7, Figure 8). An aberrant branching pattern of the superior gluteal artery was observed in 4% of the hemipelvises (right: 2, left: 1) (Figure 9).

**Figure 10:**
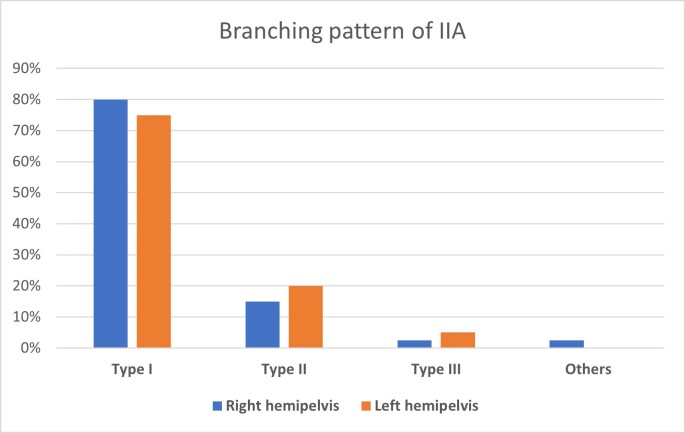
Graph showing the incidence of types of branching pattern of the internal iliac artery (IIA) (based on Adachi classification), n=80.

The branching pattern of the internal iliac artery was classified as per the modified Adachi classification. Out of 80 pelvic halves (40 right, 40 left sides), Type I arrangement was found in 80% on the right and in 75% of the left hemipelvises. Type II was observed in 15% of right and 20% of left specimens. Type III was seen in 2.5% of specimens on the right and 5% of the left pelvic halves. Type IV and Type V were not observed in any specimens (Figure 10).

## DISCUSSION

Understanding and interpreting the various branching patterns of the IIA is essential in clinical practice. Anatomists and surgeons have long noticed variations in IIA branching patterns.^6^ The IIA develops from the umbilical artery. After birth, the proximal portions of the umbilical arteries persist as the internal iliac and superior vesical arteries. The medial umbilical ligaments are formed simultaneously with the disappearance of the distal portions.^7^

Anatomical variations of the IIA were first described in 1891 by Jastchinski et al.,^8^ who observed four types of the IIA based on their analysis of the anatomy of branches of the IIA in the Polish population. Subsequently, Adachi^9^ classified the distribution pattern of the IIA from an embryological point of view into five types with eight groups. In this classification, Adachi proposed that the umbilical artery was a continuation of the main stem of the IIA, and the superior gluteal artery (SGA), the inferior gluteal artery (IGA), and the internal pudendal arteries (IPA) were principal branches of the umbilical artery. The five types referred to by Adachi are:

Type I: The SGA is the first to arise independently from the main stem, the IGA and the IPA arise from a common trunk.
Type II: The SGA and the IGA arise from a common trunk. The IPA independently arises as a major stem.
Type III: All three major branches (the SGA, the IGA, the IPA) arise separately from the IIA.
Type IV: The SGA, the IGA, and the IPA arise from a common trunk. If the SGA arises first from the common trunk and the trunk later divides into the IGA and the IPA, it is type IVa. If the IPA branches off first from the common trunk, which later divides into the SGA and the IGA, it is type IVb.
Type V: A common trunk gives rise to the SGA and the IPA, and then the IGA arises independently from the common trunk.

The frequency of occurrence of the branching pattern of the IIA and its classification based on Adachi’s types, as observed in the present study, is illustrated in Graph 1. Type I was the most common branching pattern (76%), followed by Type II (17.5%), and Type III (3.7%). These findings were in accordance with many other studies,^9-19^ as shown in Table 2.

**Table 2:** Studies based on Adachi’s classification.

Study	Type I (%)	Type II (%)	Type III (%)	Type IV (%)	Type V (%)
Adachi^9^	51.2	23.1	18.2	4.1	0.8
Braithwaite^10^	58.5	15.3	22.5	3.6	-
Yamaki et al.^11^	58.0	13.6	22.8	5.4	0.2
Al Talalwah^12^	36.1	5.3	34.8	2.3	-
Naveen et al.^13^	76.9	6.6	9.9	-	-
Ramakrishnan et al.^14^	60	4	15	-	-
Sumathilatha et al.^15^	60.6	15.8	21	-	-
Havaldar et al.^16^	52	2	34	-	2
Kumari and Gowda^17^	68	10	16	6	-
Sakthivelavan et al.^18^	69	8	20	4	-
Rajlakshmi et al.^19^	34.1	13.6	22.7	29.5	
Present study	76	17.5	3.7	-	-

It was observed that Type III variation was more common in other studies compared to ours (Table 2). This may be due to the number of specimens studied and the diversity of the population. Other rare terminal branching patterns which were observed in other studies were trifurcation into a posterior division, inferior gluteal trunk and superior vesical artery, trifurcation into posterior division, anterior division, and iliolumbar artery, or ramification into multiple branches.^20^ Type IV and V branching patterns were not observed in our study. These are considered rare branching patterns of IIA, which agrees with other Indian studies,^13-15^ who also did not encounter Type IV and V patterns.

To prevent major intraoperative complications such as accidental hemorrhage caused by incorrect interpretation of the variant arteries, knowledge of the anatomical variants of the IIA is essential during pelvic procedures. When alternative treatments fail, resulting in significant hemorrhage, bilateral IIA ligation may be the only option left.^21^ The arteries and veins supplying the organs must be ligated and transectioned after major surgical procedures such as extensive organ resection following tumors or sepsis caused by perforation of the pelvic organs.^22^

Among the variant origins of the branches of the IIA, the frequency of origin of the ilio-lumbar artery (ILA) from the main trunk of IIA was noted to be as high as 61.7% in one study.^23^ In the present study also, it was observed that the ILA took its origin from the main trunk of the IIA in 29% of the specimens. The variant origin of the ILA from the iliac artery, which can alter expected topographical relations and result in unintended hemorrhagic accidents, must be considered while performing surgical operations in the lumbar, sacral, and pelvic regions.^24,25^ In the current study, the obturator artery (OA), which exhibits lots of variation in its origin, was the next most frequently detected variant branch. Earlier studies have reported that in about 79% of cases, the OA arises from either one of the divisions of the internal iliac artery. In the remaining 21% of cases, the OA arises directly from the external iliac artery or inferior epigastric artery.^25^ Vascular connections between the obturator, internal iliac, external iliac, and inferior epigastric arteries are relatively common over the upper pubic branch and are present in about 45% of cases.^26^ The corona mortis is a vascular connection between the obturator and the inferior epigastric vessels in which either the artery or vein forms an anastomosis near the superior pubic ramus. Its clinical significance is crucial, since it can be damaged during pubic fractures or surgery.^27^ When doing several pelvic surgical procedures on both males and females, it is crucial to understand the variant origins of the OA.^28^

To prevent unanticipated problems caused by aberrant OA origin and vascular anastomoses like the corona mortis, an ilioinguinal surgical approach is advised in pelvic fractures rather than anterior access to the pelvis and acetabulum.^29^ Variations of the OA should be understood by surgeons who treat and repair pelvic hernias, superior pubic ramus, and acetabular fractures. The arrangement of the major internal iliac branches can be established by pelvic angiography, thereby avoiding unnecessary embolization or embolectomy and non-mandatory ligation during surgical procedures. Common causes of aortic access difficulties during abdominal aortic aneurysms include iliac arteries with naturally small caliber or variant anatomy.^30^ Radiologists should be aware of any vascular variations during pelvic embolization operations to reduce the risk of iatrogenic error and postoperative consequences.

## CONCLUSION

The branching pattern of the internal iliac artery was observed and classified as per the modified Adachi classification. Type I arrangement was found in 76%, Type II in 17.5%, and Type III in 3.7% of specimens. Type IV and V were not found in any specimens, which correlates with other Indian studies. Understanding the anatomical variations of the IIA and its branches is essential to minimize intraoperative blood loss and other complications during pelvic surgeries. This study contributes comprehensive knowledge regarding the vascular anatomy of IIA and its branches, which is important not only for anatomists and morphologists but also for radiologists and general and vascular surgeons.
